# Synthesis of purines and adenines containing the hexafluoroisopropyl group

**DOI:** 10.3762/bjoc.16.224

**Published:** 2020-11-11

**Authors:** Viacheslav Petrov, Rebecca J Dooley, Alexander A Marchione, Elizabeth L Diaz, Brittany S Clem, William Marshall

**Affiliations:** 1 Chemours Co. Fluoroproducts, Chemours Discovery Hub N2-136S, 201 Discovery Blvd, Newark, DE 19713, USA; 2 Chemours Co. Fluoroproducts, Chemours Discovery Hub, 201 Discovery Blvd, Newark, DE 19713, USA; 3 DuPont Corporate Center for Analytical Sciences, Experimental Station, P.O. Box 80500, Wilmington, DE 19880-0500, USA

**Keywords:** adenine, caffeine, cyclic dimer of hexafluorothioacetone, hexafluoroisopropyl group, purine, 2,2,4,4-tetrakis(trifluoromethyl)-1,3-dithietane, theophylline

## Abstract

Several new derivatives of adenine, purine, and theophylline containing the (CF_3_)_2_CH group connected to a nitrogen atom of the imidazole ring were prepared by the reaction of 2,2,4,4-tetrakis(trifluoromethyl)-1,3-dithietane (**1**) with the corresponding substrates, resulting in the selective alkylation of one of the nitrogen atoms of the imidazole ring. The reaction proceeds under mild conditions in a polar solvent, giving the alkylated products in 47–78% yield. While for purine and 4- and 5-azabenzimidazole, the reaction led to a mixture of two isomers, the reaction of adenine and the corresponding 2-fluoro derivative was regioselective, resulting in the formation of only one isomer in each case. The alkylation of theophylline led to the formation of a new derivative of caffeine.

## Introduction

Despite significant progress in the last 20–30 years, the selective introduction of fluorine and polyfluoroalkyl substituents into organic molecules remains a challenging problem in the synthesis of fluorinated biologically active compounds, especially larger moieties, such as C_2_F_5_, CF(CF_3_)_2_, and CH(CF_3_)_2_. With respect to the hexafluoroisopropyl group, the methods are limited to a relatively short list: the reduction of the OH group of the C(CF_3_)_2_OH moiety (this protocol was employed in the synthesis of enantiomerically pure (*S*)-5,5,5,5',5',5'-hexafluoroleucine [1]); the Wittig reaction of Ph_3_P=C(O)C(O)OR with hexafluoroacetone, followed by the hydrogenation of the CH=C(CF_3_)_2_ unit, and the conversion of the ketoester into an amino acid [2]; and the reaction of aldehydes with either Ph_3_P/(CF_3_)_2_CCl_2_ [3] or 2,2,4,4-tetrakis(trifluoromethyl)-1,3-dithietane [4–5]. The last method was employed in the synthesis of 16,16,16,17,17,17-hexafluororetinal, as reported by the Haas group [5].

Benzimidazoles are an important class of organic materials, and many derivatives of these group are biologically active [6–9]. The benzimidazole moiety “ […] is isosteric with indole and purine nuclei, which are present in a number of fundamental cellular components and bioactive compounds. Indeed, a number of important drugs used in different therapeutic areas contain the benzimidazole ring […], such as proton pump inhibitors (omeprazole), antihypertensives (candesartan, telmisartan), antihistaminics (astemizole), antihelmintics (albendazole, mebendazole), as well as several other kinds of still investigational therapeutic agents, including antitumorals and antivirals […]” [8].

Interestingly, most fluorine-containing benzimidazoles contain the fluorinated substituent either on the aromatic ring or on a side chain connected to the aromatic ring; examples of benzimidazoles containing a fluoroalkyl group connected to a nitrogen atom of the imidazole segment are relatively rare. Known examples include benzimidazoles carrying NCF_3_ [10], NCF_2_H [11–13], NR_f_ (R_f_ = CF_2_Cl, CF_2_Br, CF_2_CFClH, CF=CFClF) [14–15], and hexafluoroisopropyl groups attached to the nitrogen atom [16]. Recently, we reported a new method for the introduction of a hexafluoroisopropyl group based on the reaction of tetrakis(trifluoromethyl)-1,3-dithietane (**1**) with the corresponding azoles [17]. Since this methodology is simple and works for a wide variety of imidazoles, including benzimidazole, we decided to explore the applicability of this method to the modification of biologically active imidazoles, such as adenine and purine derivatives. The results of this study are reported in this article.

## Results and Discussion

**Scheme 1:**
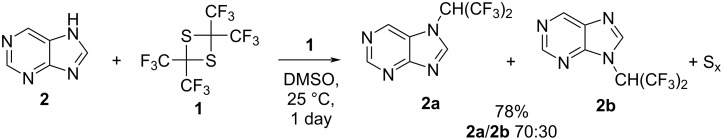
Reaction of purine (**2**) with tetrakis(trifluoromethyl)-1,3-dithietane (**1**).

Despite the presence of two electron-withdrawing nitrogen atoms in the aromatic ring, purine (**2**) was found to react with tetrakis(trifluoromethyl)-1,3-dithietane (**1**) in a manner similar to benzimidazole [17]. The reaction proceeds without catalyst in DMSO solvent at ambient temperature, leading to the formation of 7- and 9-(hexafluoroisopropyl)purine (**2a** and **2b**), respectively, along with the formation of one equivalent of elemental sulfur (Scheme 1).

**Figure 1:**
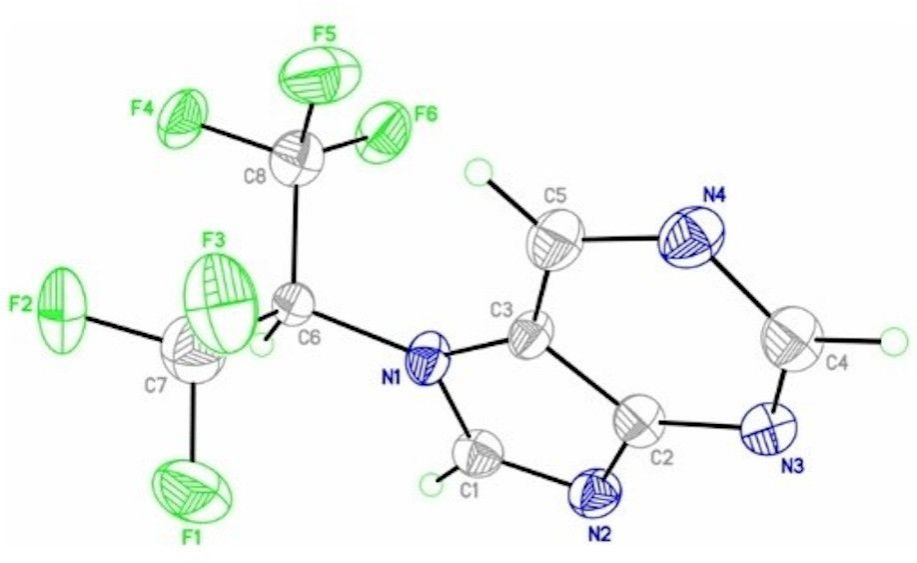
Crystal structure of **2a**, with the thermal ellipsoids drawn at 30% probability.

A crude mixture of **2a** and **2b** (ratio 70:30) was isolated in 78% yield. Since the minor isomer **2b** had a significantly higher solubility in hexane, washing of the crude mixture with hexane resulted in an enrichment of the major isomer **2a** (ratio **2a**/**2b** 95:5 after washing with hexane). Pure **2a** was isolated after recrystallization from hexane (see Experimental section), and the structure was established by X-ray diffraction (Figure 1).

**Scheme 2:**
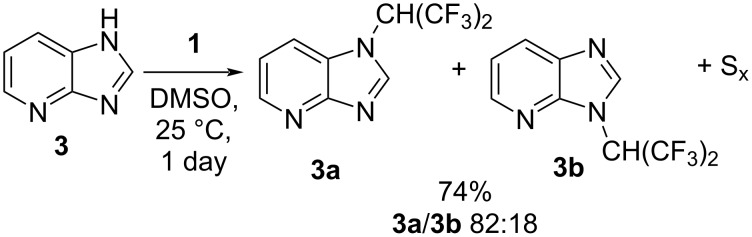
Reaction of 4-azabenzimidazole (**3**) with tetrakis(trifluoromethyl)-1,3-dithietane (**1**).

4-Azabenzimidazole (**3**) reacted with tetrakis(trifluoromethyl)-1,3-dithietane (**1**) under similar conditions, with the formation of the two isomeric products **3a** and **3b** (Scheme 2).

The major isomer **3a** was isolated in a pure form, and the structure was confirmed by X-ray diffraction. It should be pointed out that the ^19^F NMR spectra of the two isomers were very different. While the resonance corresponding to the CH(CF_3_)_2_ group in the spectrum of **3b** was a sharp and well-resolved doublet (δ = −69.95 ppm, *J*_FH_ = 6.9 Hz in CDCl_3_), the analogous resonance in the major isomer **3a** was significantly broadened (δ = −68.95 ppm, ∆√_1/2_ = 60Hz, CDCl_3_), indicating a restricted rotation around the C–N bond, likely due to the steric interaction of the CF_3_ groups of the hexafluoroisopropyl fragment with the neighboring hydrogen atom of the pyridine ring. A similar phenomenon was previously observed for *N*-(hexafluoroisopropyl)benzimidazole [17].

**Scheme 3:**
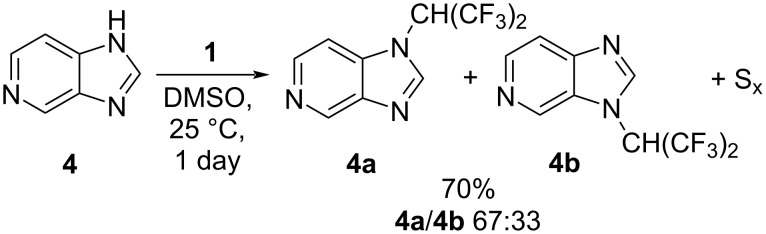
Reaction of 5-azabenzimidazole (**4**) with **1**.

5-Azabenzimidazole (**4**) reacted similarly with tetrakis(trifluoromethyl)-1,3-dithietane (**1**), which also resulted in the formation of two regioisomers, **4a** and **4b** (Scheme 3).

In this context, the isomers **4a** and **4b** were separated by column chromatography (see Experimental section), and the structure of both was established by X-ray diffraction. In the ^19^F NMR spectra of both isomers **4a** and **4b**, the resonances corresponding to the CH(CF_3_)_2_ group were significantly broadened.

**Scheme 4:**
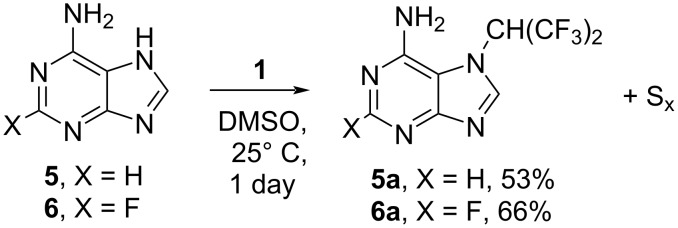
Reaction of adenine (**5**) and 2-fluoroadenine (**6**) with tetrakis(trifluoromethyl)-1,3-dithietane (**1**).

While all attempts to involve guanine in the reaction with **1** failed (DMSO, 25–70 °C, 4 d), the reaction of adenine (**5**) or 2-fluoroadenine (**6**) with tetrakis(trifluoromethyl)-1,3-dithietane (**1**) was found to proceed under mild conditions (DMSO, 25 °C, 1 d), leading (unexpectedly) to the formation of only one isomer (**5a** and **6a**, respectively) in each case (Scheme 4).

Another surprising feature of these reactions was the selective alkylation of the nitrogen atoms of the imidazole ring rather than the amino group. This is in sharp contrast to the report by Ishikawa and Kitazume that the reaction of anilines with tetrakis(trifluoromethyl)-1,3-dithietane (**1**) results in the formation of the corresponding imines of hexafluoroacetone [18]. Unfortunately, we were not able to confirm the structures by X-ray diffraction, and the structural assignment for the compounds **5a** and **6a** relies solely on NMR data. In both cases, the data were consistent with the structures **5a** or **6a**, carrying NH_2_ and CH(CF_3_)_2_ groups on the same side of the molecule. Indeed, in the ^19^F NMR spectra of **5a** and **6a**, the resonance corresponding to CH(CF_3_)_2_ was significantly broadened (**5a**: ∆√_1/2_ = 150 Hz; **6a**: ∆√_1/2_ = 50 Hz), and in the ^1^H NMR spectrum of **6a**, the ^1^H nuclei of the NH_2_ group were observed as two separate resonances, with significantly different chemical shifts (δ = 1.66 (br. s) and 5.98 ppm (1H, br. s) in CDCl_3_ solvent, while in DMSO-*d*_6_, only one broad resonance (δ = 7.69 ppm, 2H, br. s) was observed for the NH_2_ group. We believe that these observations are consistent with the restricted rotation of the CH(CF_3_)_2_ group because of the proximity to the amino group.

**Scheme 5:**
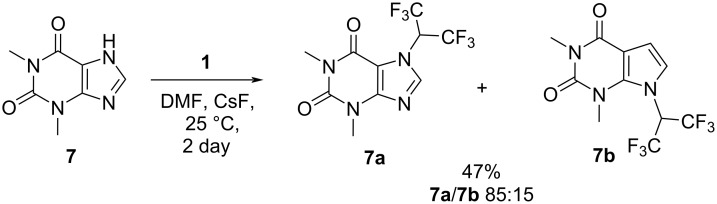
Reaction of theophylline (**7**) with tetrakis(trifluoromethyl)-1,3-dithietane (**1**).

Theobromine or caffeine both were found to be inert towards tetrakis(trifluoromethyl)-1,3-dithietane (**1**) – even at elevated temperature (65 °C, 4–6 d, DMSO or DMF) or in the presence of a catalyst (CsF, DMSO, 25 °C, 2 d), but the structurally similar theophylline (**7**) reacted with **1** in the presence of CsF catalyst, producing a mixture of the two isomers **7a** and **7b**, with a significant predominance of **7a** (Scheme 5).

**Figure 2:**
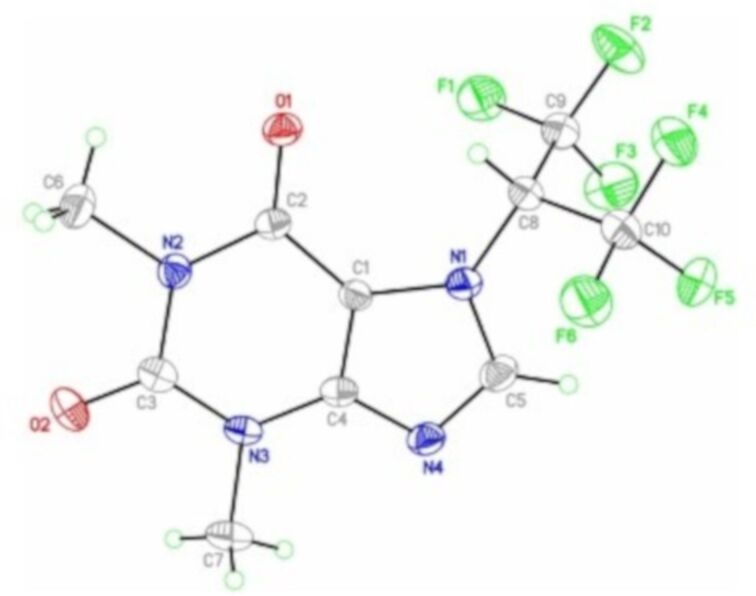
Crystal structure of **7a**, with the thermal ellipsoids drawn at 30% probability.

The major isomer **7a** was isolated by recrystallization from hexane, and the structure was unambiguously established by X-ray diffraction (Figure 2).

Exchange broadening of the resonances of the (CF_3_)_2_CH group was not observed in the ^19^F NMR spectra of **7a** and **7b**.

Compound **7a** can be viewed as a caffeine analogue, modified by the attachment of two CF_3_ groups to the methyl group. This is the first example of an introduction of a fluoroalkyl group bigger than CF_3_ or CF_2_H into a theophylline (**7**) molecule, since known examples of fluorinated caffeine derivatives are limited to 7-trifluoromethyl caffeine, claimed in the patent literature [19–20] and 7-difluoromethyl caffeine, prepared by the insertion of difluorocarbene into the N–H bond of theophylline (**7**) [11,21–22]. Similar to the reaction of tetrakis(trifluoromethyl)-1,3-dithietane (**1**) and theophylline (**7**, Scheme 5), this process also led to the formation of two regioisomers, but interestingly, the ratio of the 7-CF_2_H- and 9-CF_2_H-isomers varied broadly from 1:2 [22] to 3:2, depending on the reaction conditions and the source of difluorocarbene [11].

### Mechanism, regiochemistry, and rotation barriers of the CH(CF_3_)_2_ group

**Scheme 6:**
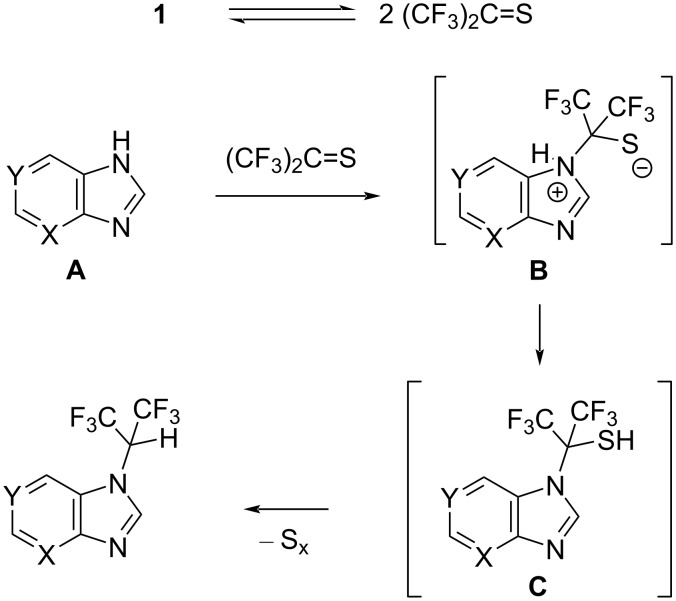
Probable mechanism of the reaction of tetrakis(trifluoromethyl)-1,3-dithietane (**1**) with compounds **2**–**7**.

The mechanism of the reaction of tetrakis(trifluoromethyl)-1,3-dithietane (**1**) with the azoles **2**–**7** is not entirely clear. Although there are two similar reactions reported in the literature (the reaction of **1** with anilines [18] and azoles [17]), no satisfactory mechanism was suggested for these reactions. Recently, it was demonstrated that the majority of the reactions of **1** can involve monomeric hexafluorothioacetone (HFTA) as long as the reaction is carried out in a polar and nucleophilic solvent, such as DMF or DMSO [23]. One can speculate that an alternative mechanism may involve the interaction of monomeric HFTA and imidazole. In this case, the reaction can proceed through the attack of a nitrogen atom of the imidazole ring at the carbon atom of the C=S bond, leading to the formation of the zwitterion **B** (Scheme 6).

The transfer of a proton from the nitrogen atom to the sulfur atom leads to the formation of thiol **C**, which can lose sulfur, giving rise to the final product. Because of the unsymmetrical nature of substrate **A**, the attack on the C=S bond of HFTA proceeds by either nitrogen atom of the imidazole ring, resulting in the formation of two isomers for compounds **2**–**4** and **7**. The highly selective reaction of adenines **5** and **6** may reflect a significantly higher nucleophilicity of the nitrogen atom of the imidazole ring versus that of the NH_2_ group.

It should be pointed out that the mechanism depicted in Scheme 6 is highly speculative since the nature of the last step (the conversion of intermediate **C** into the final product, with extrusion of elemental sulfur) is not well understood and may be more complex, involving additional steps. The mechanism clarification of the reaction of tetrakis(trifluoromethyl)-1,3-dithietane (**1**) with azoles (and anilines) does require further investigation.

As was mentioned above, the broadening of the resonances corresponding to the CF_3_ group as observed in the ^19^F NMR spectra of derivatives **3a**, **4a**, and **4b** at ambient temperature is likely to be related to a restricted rotation around the C–N bond as a result of the steric interaction between the fluorine atoms of the (CF_3_)_2_CH group and a neighboring hydrogen of an aromatic ring. Indeed, a similar phenomenon was observed for benzimidazole carrying a CH(CF_3_)_2_ group at a nitrogen atom of the imidazole ring [17]. Another argument in favor of this explanation is the absence of any evidence for the restricted rotation in the isomer **3b** (Scheme 2), which has a nitrogen atom in the β-position relative to the CH(CF_3_)_2_ group (see Table 1), while signal broadening was clearly present in the ^19^F NMR spectra of both isomers **4a** and **4b**. All these data are consistent with the observed restricted rotation of the (CF_3_)_2_CH group in compounds **5a** and **6a** (see Table 2) because of the presence in the β-position relative to the CH(CF_3_)_2_ unit of the amino group, which is substantially bigger than a hydrogen atom.

**Table 1:** Reaction products, conditions, yield, ratio of isomers, and melting points for the new materials.

compound	solvent (cat.)	*T*, °C (time, d)	yield, %	ratio of isomers (crude product)	mp, °C (crude product)	mp, °C (recrystallized from hexane)

**2a**	DMSO	25 (1)	78	**2a**/**2b** 70:30	158–167	–
**2b**						**2b**, 122–123
**3a**,**b**	DMSO	25 (1)	74	**3a**/**3b** 82:18	137–163	**3a**, 164–165
**4a**,**b**	DMSO	25 (1)	70	**4a**/**4b** 67:33	–	**4a**, 157–158 **4b**, 110–111
**5a**	DMSO	25 (1)	53	one isomer	212–214 (dec.)	**5a**, 214–216 (dec.)
**6a**	DMSO	25 (1)	66	one isomer	–	**6a**, 220–222
**7a**,**b**	DMF (CsF)	25 (4)	47	**7a**/**7b** 85:15	86–88	**7a**, 95–95.5 (purity 98%)

**Table 2:** Enthalpy and entropy of activation for the rotameric interconversion of a subset of hexafluoroisopropyl azoles.

compound	Δ*H*^‡^, major to minor (kcal⋅mol^−1^)	Δ*S*^‡^, major to minor (cal⋅mol^−1^ K^−1^)	Δ*H*^‡^, minor to major (kcal⋅mol^−1^)	Δ*S*^‡^, minor to major (cal⋅mol^−1^ K^−1^)	population of the major rotamer at 298 K

**3a**	11.6	−5.38	11.1	−5.90	0.691
**4a**	12.0	−4.12	11.5	−3.67	0.760
**4b**	11.6	−5.91	10.6	−7.76	0.680
**6a**	12.0	−3.72	9.79	−7.09	0.862

### Dynamic NMR spectroscopy

**Figure 3:**
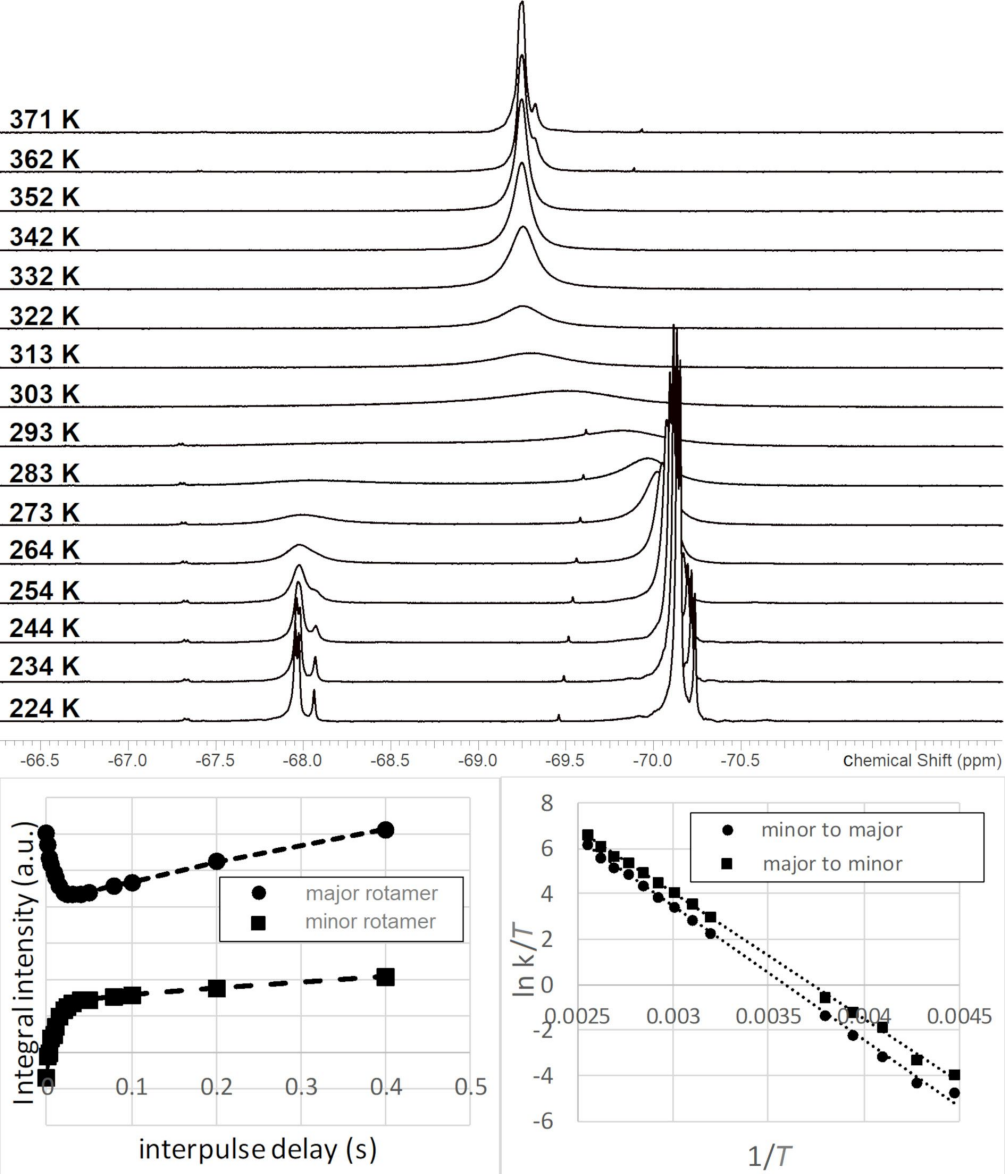
Top: ^19^F NMR spectra of **3a** acquired over a sample temperature range of 223–373 K. Left: Fitted plot of integrated intensity vs interpulse delay time from the experiment inverting the minor rotamer of **3a** at 253 K. Right: Eyring plot of the interconversion of the rotamers of **3a**.

The broadening of resonances corresponding to the hexafluoroisopropyl moieties in the ^19^F NMR spectra described above was observed at near-ambient temperatures and at resonance frequencies from 376 to 564 MHz, consistent with a slow exchange between rotameric states on the NMR timescale under these conditions. The activation parameters of the hindered rotation were obtained for a subset of these compounds, **3a**, **4a**, **4b**, and **6a**, by NMR experiments similar to those used in recent studies [24]. In brief, solutions of each compound were made in *N*,*N*-dimethylformamide-*d*_7_. A series of ^19^F NMR spectra were acquired at subambient temperature: both simple one-dimensional spectra and selective inversion spectra, in which the resonance corresponding to one rotamer was selectively inverted, allowed to interconvert with the other site for a variable period and then converted to observable magnetization with an excitation pulse. The former experiments allowed an assay of the population of each site as a function of the temperature, which enabled an extrapolation of the population differences at an elevated temperature (via the van ‘t Hoff equation). The latter experiments provided information directly on the kinetics of the interconversion between the sites. A series of spectra were then acquired at elevated temperature; here, the kinetics of the interconversion between the sites could be derived directly from the linewidth. Figure 3 illustrates the spectra acquired over a range of temperatures using compound **3a** as an example. With the rate constants of the interconversion between the rotamers in hand, an Eyring plot was generated, and the enthalpy and entropy of activation were derived (Table 2).

**Figure 4:**
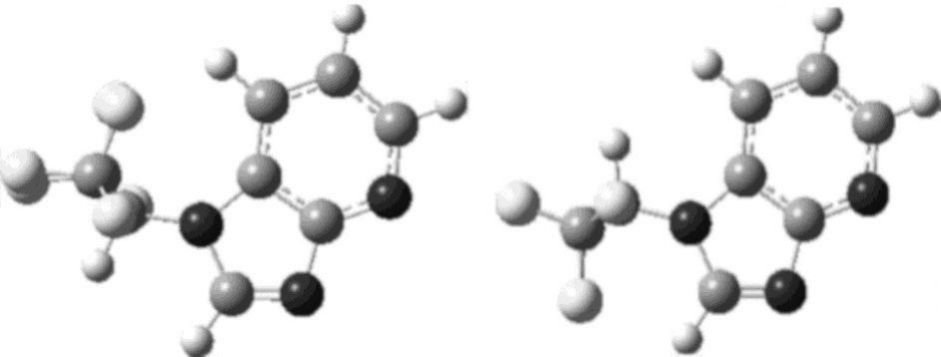
DFT-optimized structures of the two rotamers of **3a**. Left: Lower-energy rotamer. Right: Higher-energy rotamer.

The structural identification of the major and minor rotamers was not attempted by NMR, but in silico investigations of **3a** supported the intuitive notion that the rotamer of lower energy was that with the CF_3_ moieties nearer the five-membered ring of the azabenzimidazole unit, and the rotamer of higher energy was that with the CF_3_ moieties near the six-membered ring (see Figure 4). Density functional theory calculations predict an enthalpy difference of 0.48 kcal⋅mol^−1^ between the two rotamers. By comparison, Δ*H* between the two rotamers was obtained experimentally by fitting the van ‘t Hoff equation to the population differences observed in the ^19^F NMR spectra between 224 and 264 K. 0.89 kcal⋅mol^−1^ is in tolerable agreement with the computation.

Comparing **3a**, **4a**, **4b**, and **6a** in Table 2, the enhanced imbalance in the population of the rotamers of **6a** is notable and consistent with the amino moiety imposing a greater steric hindrance. The differences among the other compounds are more modest. The small entropy of activation is consistent with a simple rotation over a steric barrier (with minimal contribution from such forces as hydrogen bonding or solvent interactions), and the relative enthalpy of activation for each compound (major to minor vs minor to major) are roughly similar to the difference in total enthalpy for the two rotamers for each compound.

## Experimental

GC and GC–MS analyses were carried out on a HP-6890 instrument using a HP FFAP capillary column and either TCD (GC) or mass-selective detectors (GS–MS), respectively. Purine (**2**), 4-aza- and 5-azabenzimidazole (**3** and **4**), adenine (**5**), theophylline (**7**), dry DMF, and DMSO were obtained from commercial source (Sigma-Aldrich) and used without further purification. CsF (Sigma-Aldrich) was dried in vacuum and stored and handled inside a glove box. Compound **1** was prepared according to a literature procedure [24], purity 96–98% and was used without further purification. The purity of the new materials, determined by GC and NMR spectroscopy, was at least 99%, unless stated otherwise.

### Standard NMR experiments

The NMR data presented in Table 2 and Table 3 were derived from spectra acquired on a Bruker Neo spectrometer with a 9.4 T magnetic field, equipped with a 5 mm ^15^N,^77^Se, ^31^P {^19^F,^1^H} nitrogen cryoprobe. Chemical shifts are referenced to internal tetramethylsilane or trichlorofluoromethane. The spectra were acquired at 298 K.

**Table 3:** NMR and MS data for the compounds **2a**,**b**, **3a**,**b**, **4a**,**b**, **5a**, **6a**, and **7a**,**b**.

compound	^1^H NMR (in CDCl_3_, δ, ppm; *J*, Hz)	^19^F NMR (in CDCl_3_, δ, ppm; *J*, Hz)	^13^C NMR (δ, ppm; *J*, Hz)^a^	MS (*m*/*z*)^b^

**2a**	5.55 (1H, sept, 6.5), 8.40 (1H, s), 9.08 (1H, s), 9.28 (1H, s)	−68.75 (d, 6.6)	–	270 (M^+^, C_8_H_4_F_6_N_4_^+^)
**2b**	6.08 (1H, sept, 7.0), 8.33 (1H, s), 9.09 (1H, s), 9.30 (1H, s)	−69.75 (d, 7.0)	55.07 (sept, 33.7), 120.51 (q, 283.8), 133.87, 141.75 (sept, 1.5), 149.83, 151.43, 153.80	270 (M^+^, C_8_H_4_F_6_N_4_^+^)
**3a**	5.63 (1H, sept, 6.7), 7.40 (1H, dd, 8.4, 4.7), 7.89 (1H, d, 8.4), 8.37 (1H, s), 8.70 (1H, d, 4.7)	−68.95 (br. s, ∆√_1/2_ = 60 Hz)	52.50 (br. s), 118.99, 119.61, 121.61 (q, 287.00), 144.00, 146.01, 159.81, 205.22	269 (M^+^, C_9_H_5_F_6_N_3_^+^)
**3b**	6.18 (1H, sept), 7.40 (1H), 8.25 (1H), 8.37 (1H), 8.50 (1H)	−69.95 (d, 6.9)	–	–
**4a**	5.46 (sept, 6.7), 7.41 (1H, d, 5.7), 8.15 (1H, s), 8.61 (1H, d, 5.7), 9.24 (1H, s)	−69.15 (br. s)	58.38 (sept, 32.8), 115.74, 120.78 (q, 284.4), 131.28, 132.90, 143.65, 144.00, 148.35	269 (M^+^, C_9_H_5_F_6_N_3_^+^)
**4b**	5.46 (sept, 6.7), 7.41 (1H, d, 5.7), 8.15 (1H, s), 8.61 (1H, d, 5.7), 9.24 (1H, s)	−69.08 (br. s)	58.10 (sept, 33.2), 104.64, 120.77 (q, 284.4), 138.78, 139.97, 142.11, 144.06, 144.09	–
**5a**	5.67 (2H, br. s), 5.95 (1H, sept, 7.2), 8.02 (1H, s), 8.48 (1H, s); DMSO-*d*_6_: 7.23 (1H, sept, 7.2 Hz), 7.64 (2H, s), 8.27 (1H, s), 8.42 (1H, s)	−69.08 (br. s, ∆√_1/2_ > 150 Hz); DMSO-*d*_6_: −69.88 (br. s, ∆√_1/2_ > 150 Hz)	55.03 (sept, 33.0), 117.93, 121.44 (q, 282.0), 138.87, 150.41, 154.27, 156.20^c^	285 (M^+^, C_8_H_5_F_6_N_5_^+^)
**6a**	1.66 (1H, br. s), 5.81 (1H, sept, 7.2), 5.98 (1H, br. s), 7.98 (1H, s); DMSO-*d*_6_: 6.48 (1H, sept, 7.1), 7.69 (2H, br. s), 7.96 (1H, s)	−69.84 (6F, br. s, ∆√_1/2_ = 50 Hz), −49.15 (1F, br. s); DMSO-*d*_6_: −69.4 (6F, br. s, ∆√_1/2_ = 50 Hz), −50.42 (1F, br. s)	59.70 (sept, 32.0), 117.21, 121.51 (q, 283.8), 139.27, 151.88 (d, 20.4), 158.55 (d, 21.70), 159.68 (d, 205.9)^c^	303 (M^+^, C_8_H_4_F_7_N_5_^+^)
**7a**	3.40 (3H, s), 3.60 (3H, s), 6.74 (1H, sept, 7.0), 7.85 (1H, s)	−70.00 (d, 7.0)	–	330 (M^+^, C_10_H_8_F_6_N_4_O_2_^+^)
**7b**	3.39 (3H, s), 3.59 (3H, s), 4.68 (1H, sept, 7.8), 7.80 (1H, s)	−65.02 (d, 7.8)	–	–

^a13^C{^1^H} in CDCl_3_, unless stated otherwise. ^b^Mixture of isomers, solution in acetone. ^c^In DMSO-*d*_6_ as a solvent.

### Dynamic NMR experiments

The dynamics of rotamer interconversion were studied on a Bruker AVIIIHD spectrometer with a 9.4 T magnetic field, equipped with a 10 mm F{H} probe suitable for high- and low-temperature operation. The sample temperature was calibrated against neat ethylene glycol and neat methanol standards. Selective inversion experiments at subambient temperature effected the selective inversion with iBURP shapes [25]. The spin–lattice relaxation time *T*_1_ for each site was determined with nonselective inversion recovery experiments. The rate constant for the interconversion between the rotamers at each temperature was determined using numerical integration, minimizing the square of residuals, with the obtained *T*_1_ relaxation time as an input to the model. The linewidths at elevated temperature were determined using the peak fitting utility in ACDLabs Spectrus Processor software (v. 2018.1.1).

### X-ray diffraction

X-ray data for **7a**, **2a**, **3a**, **4a**, and **6a** were collected at −100 °C using a Bruker APEX-II CCD system equipped with a sealed tube molybdenum source and a graphite monochromator. The structures were solved and reﬁned using the SHELXTL software package [26], reﬁnement by full-matrix least squares on F2, scattering factors from [25]. Crystallographic data (excluding structure factors) for the structures in this paper have been deposited with the Cambridge Crystallographic Data Center as supplementary publication nos. CCDC #1998565−1998569.

### Computational chemistry

Computational optimization of **8a** and **8b** structures was performed with Gaussian 9 software. Density functional theory at the B3LYP level, with a 6-311g++ basis set (Co phase) was employed.

### Reaction of purine (**2**) and **1**

A mixture of 1.2 g (0.01 mol) of purine (**2**), 3 g (0.08 mol) of **1**, and 15 mL of DMSO was agitated for 24 h at ambient temperature. The precipitated sulfur was filtered and dried on a filter (isolated: 0.25 g, 78%). The filtrate was poured in 300 mL of water, and the mixture was extracted with hexane (50 mL × 2). The hexane layer was dried, and the solvent was removed under vacuum to leave 0.6 g of a white solid, which was found to be the 99% pure minor isomer **2b** (mp 122–123 °C after the second crystallization from hexane; this sample was used for X-ray diffraction studies). The rest of the reaction mixture was extracted with DCM (50 mL × 2). The DCM layer was washed with water (200 mL × 3) and dried, and the solvent was removed under vacuum to leave 1.5 g of a crude orange solid, which was found to be a mixture of **2a** and **2b** in the ratio 70:30 (GC, NMR), mp 158–167 °C. Total yield of **2a**,**b**: 78%.

A small sample of a mixture of **2a**,**b** was washed with cold hexane on a ceramic filter to give a material with a **2a**,**b** ratio of 95:5. Recrystallization of this sample from hexane gave the isomer **2b** (purity 99%, by NMR), and this sample was used for an X-ray diffraction experiment. The reaction conditions, mass spectrometry, and NMR data are given in Table 1 and Table 3.

### Reaction of 4-azabenzimidazole (**3**) and **1**

A mixture of 1.2 g (0.01 mol) of 4-azabenzimidazole (**3**), 2 g (0.06 mol) of **1**, and 15 mL of DMSO was agitated at ambient temperature for 24 h. The precipitated sulfur was filtered, washed with hexane, and dried on a filter (isolated: 0.25 g, 78%). The reaction mixture was poured into 500 mL of water, and the solids were extracted with a 1:1 mixture of hexane/dichloromethane (50 mL × 2), washed with water (100 mL × 2), dried with MgSO_4_, and filtered. The solvent was removed under reduced pressure to give 2 g (74%) of the crude product, which was found to be a mixture of two isomers (ratio 82:18, by NMR), mp 137–163 °C.

A small sample of this material was recrystallized from hot hexane to give a sample of the major isomer **3a** (purity 99.8%, mp 164–165 °C), which was used for an X-ray diffraction experiment. The reaction conditions, mass spectrometry, and NMR data are given in Table 1 and Table 3.

### Reaction of 5-azabenzimidazole (**4**) and **1**

A mixture of 1.2 g (0.01 mol) of 5-azabenzimidazole (**4**), 2 g (0.06 mol) of **1**, and 15 mL of DMSO was agitated at ambient temperature for 24 h. The precipitated sulfur was filtered. The reaction mixture was poured in 500 mL of water, and the solids were filtered off to give 1.9 g (70%) of a crude product, which was found to be a mixture of the isomers **4a** and **4b** in a ratio of 67:33 (by NMR).

Pure isomers were isolated using column chromatography (Biotage instrument, hexane/ethyl acetate gradient, 100 mL/min, cartridge SNAP 340 g). The structure of each isomer was determined by X-ray diffraction. The reaction conditions, mass spectrometry, and NMR data are given in Table 1 and Table 3.

### Reaction of adenine (**5**) and 2-fluoroadenine (**6**), respectively, and **1**

A mixture of 0.01 mol of the corresponding adenine (1.35 g of **5** or 1.53 g of **6**), 2 g (0.06 mol) of **1**, and 15 mL of DMSO was agitated at ambient temperature for 24 h. The precipitated sulfur was filtered and dried on a filter (isolated: 0.25 and 0.30 g, 78–94%, respectively). The reaction mixture was poured in 500 mL of water, and the solids were filtered off, washed with water (100 mL × 2), and dried on a filter. The crude material was washed with cold hexane and dried to give 1.51 g of **5a** (yield 53%, purity 98%) and 1.99 g of **6a** (yield 66%, purity 97%). Pure samples (>99%, by GC and NMR) of **5a** and **5b** were obtained by recrystallization of small sample from hexane. The reaction conditions, mass spectrometry, and NMR data are given in Table 1 and Table 3.

### Reaction of theophylline (**7**) and **1**

A mixture of 1.8 g (0.01 mol) of theophylline (**7**), 2 g (0.012 mol) of **1**, 0.5 g (0.003 mol) of dry CsF, and 15 mL of DMF was agitated for 4 d at ambient temperature. The reaction mixture was filtered to remove precipitated sulfur and the catalyst. The filtrate was poured in 300 mL of water, and the mixture was extracted by a dichloromethane/hexane mixture (3:1, 50 mL × 2). The organic layer was dried with MgSO_4_, filtered, and the solvent was removed under reduced pressure to give 1.5 g of a slightly yellow solid. This was redissolved in 20 mL of 1:1 mixture of dichloromethane/hexane and kept at −15 °C. A yellow precipitate (0.1 g) was filtered off, and the solvent was removed to leave 1.2 g of the crude product (mp 86–88 °C), which was found to be a mixture of the two isomers **7a** and **7b** in a ratio of 85:15 (purity 98%, yield 47%). A small sample of this material was recrystallized from hot hexane to give a sample of **7a** (mp 95 °C, purity 99%), which was used for an X-ray diffraction experiment. The identity of a single crystal and the bulk of the material was confirmed by powder diffraction data and compared to simulated powder diffraction data from the single crystal diffraction experiment. The reaction conditions, mass spectrometry, and NMR data are given in Table 1 and Table 3.
